# Die COVID-19-Pandemie veränderte nicht die Zahl, aber die Art psychiatrischer Notfälle: Versorgungsdaten aus Vergleichszeiträumen von 2019 und 2020

**DOI:** 10.1007/s00115-020-00973-2

**Published:** 2020-07-24

**Authors:** Lilian Aly, Rebecca Sondergeld, Patricia Hölzle, Andreas Frank, Benjamin Knier, Esther Pausch, Michael Dommasch, Hans Förstl, Bastian Fatke

**Affiliations:** 1 Klinik und Poliklinik für Psychiatrie und Psychotherapie, Klinikum rechts der Isar, Technische Universität München, München, Deutschland; 2 Klinik und Poliklinik für Neurologie, Klinikum rechts der Isar, Technische Universität München, München, Deutschland; 3 Klinik und Poliklinik für Innere Medizin I, Klinikum rechts der Isar, Technische Universität München, München, Deutschland

## Hintergrund

Die neuropsychiatrischen Auswirkungen von COVID-19 reichen von den biologischen Effekten des neurotropen Virus [8] über Furcht vor einer Infektion zu den indirekten psychosozialen Konsequenzen von Isolation und finanziellen Einbußen [6] bis hin zu Belastungen des Gesundheitspersonals [5]. Die aktuelle Literatur weist neben der generellen Belastung auch auf eine besondere Gefährdung von Menschen mit psychischen Vorerkrankungen hin, die aufgrund ihrer Vulnerabilität und Lebenslage die Folgen der Krise noch stärker zu spüren bekommen [9, 10]. Nach unserem ersten klinischen Eindruck hat sich das Anforderungsprofil im Bereich psychiatrischer Notfälle während der Krise verändert [2].

Ziel dieser Arbeit war die quantifizierte Erfassung von PatientInnen mit und ohne psychische Erkrankungen, die sich als Notfälle während der COVID-19-Pandemie im Vergleich zum Vorjahr in der zentralen Notaufnahme vorstellten. Qualitativ wurden zudem spezifische Belastungsprofile konsiliarisch mitbetreuter PatientInnen im Zusammenhang mit COVID-19 ausgewertet.

## Datenerhebung und Auswertung

Wir untersuchten die Zahl der Vorstellungen in der zentralen Notaufnahme an unserem Klinikum seit Einführung der Ausgangsbeschränkungen in Bayern (Untersuchungszeitraum: 21.03.2020 bis 01.05.2020).

Jede/r PatientIn wurde nach dem validierten Manchester-Triage-System (MTS) von geschultem Pflegepersonal erstuntersucht [1] und die starken psychischen Belastungen entsprechend systematisch eingeteilt in „psychische Erkrankung“, „auffälliges Verhalten“, „betrunkener Eindruck“ oder „Überdosierung und Vergiftung“.

Abhängig von der Ersteinschätzung wurde ein Teil dieser Notfälle in der Folge ärztlich untersucht. Dabei wurden ICD-10-Diagnosen von ÄrztInnen beteiligter Fachrichtungen (Innere Medizin, Neurologie, Psychiatrie) vergeben.

Wir verglichen die gesamte wöchentliche Anzahl aller Vorstellungen und den Anteil von Fällen mit psychischer Belastung jeweils mit einem Referenzzeitraum des Vorjahres (23.03.2019 bis 03.05.2019).

In der Gruppe der ärztlich untersuchten PatientInnen mit ICD-10-Diagnosen verglichen wir die gesamte wöchentliche Anzahl der PatientInnen und den Anteil an psychiatrischen Hauptdiagnosen (ICD-10 F0–6) mit demselben Referenzzeitraum (jeweils ungepaarter, zweiseitiger t‑Test nach Mann-Whitney-U)*. *Die Berechnung der Statistik erfolgte in GraphPad Prism 7.0e.

Ferner untersuchten wir im selben Zeitraum 2020 alle psychiatrischen Konsilanforderungen aufgrund COVID-assoziierter psychischer Belastungen im gesamten Klinikum. Als COVID-assoziiert wurden alle Konsile bewertet, in denen in retrospektiver Analyse eine explizite Belastung durch die COVID-19-Pandemie erwähnt und inhaltlich begründet wurde. Der Anteil von Suizidversuchen unter konsiliarisch betreuten PatientInnen mit und ohne Angabe einer COVID-19-Belastung wurde mittels zweiseitigem χ^2^-Test ausgewertet.

## Höherer Anteil psychischer Probleme in der zentralen Notaufnahme

**Figure 1:**
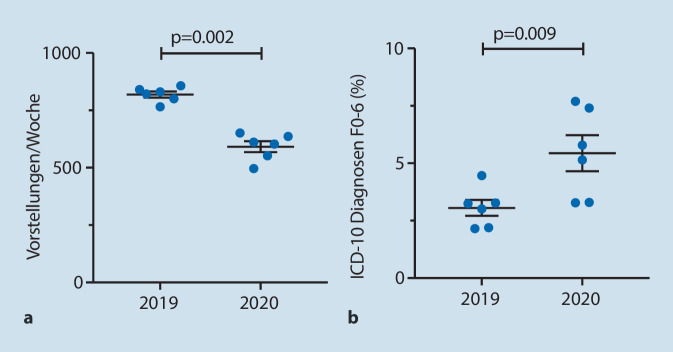

Die Zahl aller PatientInnen, die sich als Notfälle in unserem Klinikum vorstellten, war im Untersuchungszeitraum 2020 mit insgesamt 3549 niedriger als im Vergleichszeitraum 2019 mit 4913 (Abb. 1a). Im Vergleich zum Vorjahr stieg der Anteil der Fälle von MTS-Indikatoren mit Hinweisen auf psychische Belastung.

Die Gesamtzahl der ärztlich untersuchten und ICD-kodierten PatientInnen der Notaufnahme nahm ebenfalls im Vergleich zum Vorjahr im Trend ab (2019: *n* = 1846; 2020: *n* = 1112). Die absolute Zahl der PatientInnen mit einer psychiatrischen Diagnose blieb dabei im Vergleich zum Vorjahr unverändert (*n* = 57). Damit erhöhte sich im Jahresvergleich der Anteil an psychiatrischen ICD-Hauptdiagnosen (ICD-10: F0–6) von 3 auf 5 % signifikant (Abb. 1b). In Bezug auf die Verteilung psychiatrischer ICD-10-Hauptdiagnosegruppen fand sich im Vergleich zum Vorjahr kein signifikanter Unterschied (2019/2020 ICD-10 F0 *n* = 2/4; F1 15/16; F2 8/7; F3 3/5; F4 29/22; F5 0/1; F6 0/2).

## Veränderte konsiliarische Anforderungen

Im Untersuchungszeitraum 2020 hatten 49 von insgesamt 231 psychiatrischen Konsilfällen (21 %) einen Bezug zu COVID-19. Bei diesen zeichneten sich folgende Problembereiche und Belastungsprofile ab:

- Akute Verwirrtheitszustände bei COVID-19-positiven Patienten: Sieben männliche Patienten, Durchschnittsalter 60 Jahre, entwickelten im Rahmen der COVID-19-Pneumonie ein Delir, das aufgrund seiner Ausprägung zur psychiatrischen Vorstellung führte. Vier von ihnen litten an schweren somatischen Vorerkrankungen, drei an einer Suchterkrankung.
- Besuchsverbot im Klinikum als Ursache einer deutlichen psychischen Belastung: 14 PatientInnen, sieben von ihnen weiblich, gaben an, unter Einsamkeit und Isolation als Folge des eingeschränkten Kontakts zu Angehörigen zu leiden. Dies betraf fünf COVID-19-positive und neun COVID-19-negative PatientInnen. Zu dieser Gruppe zählten überwiegend PatientInnen mit multiplen somatischen Vorerkrankungen, schwerem Krankheitsverlauf und langem Aufenthalt im Krankenhaus.
- Angst vor einer Infektion mit COVID-19: Vier PatientInnen ohne Hinweise auf eine Infektion gaben an, dass sich ihre psychische Verfassung aufgrund von Angst vor einer COVID-19-Infektion substanziell verschlechtert habe. Das Durchschnittsalter lag bei 51 Jahren, alle gehörten aufgrund somatischer Vorerkrankungen zur Risikogruppe für einen schwereren Verlauf einer potenziellen COVID-19-Infektion.
- Belastung durch Ausgangsbeschränkung: Zwölf PatientInnen waren im Zusammenhang mit der Ausgangsbeschränkung psychisch belastet. In der Gruppe fand sich bei sieben Fällen eine positive Suchtanamnese, bei fünf bestand eine psychische Erkrankung in der Anamnese, vier PatientInnen hatten vor der psychiatrischen Vorstellung einen Suizidversuch unternommen.
- Sozioökonomische Probleme infolge der Pandemie: Acht PatientInnen gaben an, durch drohenden Arbeitsplatzverlust oder finanzielle Einbußen infolge der Ausgangsbeschränkungen und Betriebsuntersagungen unter einer massiven Belastung zu leiden. Das Durchschnittsalter lag bei 47 Jahren. Auch in dieser Gruppe hatte mit drei PatientInnen ein relativ hoher Anteil einen Suizidversuch unternommen.
- Reduzierte psychiatrische Versorgung: Vier PatientInnen mit psychischen Vorerkrankungen, drei von ihnen mit zusätzlichen Suchterkrankungen, beklagten eine verschlechterte psychische Verfassung aufgrund fehlender Möglichkeit einer ausreichenden stationären oder ambulanten psychiatrischen Versorgung.

## Hohe Gefährdung

Insgesamt elf von 49 PatientInnen mit COVID-assoziierten Problemen unternahmen einen Suizidversuch. Wir verglichen den Anteil an Suizidversuchen der PatientInnen, die im Zeitraum der Erhebung eine Belastung durch COVID-19 angaben, mit dem Anteil an Suizidversuchen unter PatientInnen, die ohne COVID-Belastung im gleichen Zeitraum konsiliarisch psychiatrisch betreut wurden. Im Kollektiv COVID-belasteter PatientInnen war der Anteil an Suizidversuchen mit 22 % im Vergleich zu PatientInnen ohne Anamnese einer COVID-Belastung (Suizidversuche bei 6 %) signifikant erhöht (χ^2^, *p* = 0,001).

## Diskussion und Limitationen

Während der COVID-19-Pandemie fand sich eine deutlich reduzierte Gesamtzahl notfallmäßiger Krankenhausvorstellungen im Vergleich zum Vorjahr. Auch wenn diesbezüglich bisher flächendeckende Studiendaten fehlen, deckt sich dieser Befund mit Stellungnahmen verschiedener Fachgesellschaften [3, 4]. Ein verändertes Beschwerdeprofil und aktuelle Belastungen zeigten sich in der untersuchten Gruppe in Form eines erheblichen Anteils an Suizidversuchen bei COVID-19-belasteten Patienten. Hinweise hierfür fanden sich bereits in Berichten von COVID-19-assoziierten Suiziden [7].

Der Anteil der COVID-assoziierten Suizidversuche an der Gesamtzahl der Konsile erscheint mit 21 % hoch. Die absolute Anzahl ist mit 49 Fällen jedoch zu gering, um statistisch gesicherte Rückschlüsse über einzelne Subgruppen zu erlauben. Darüber hinaus wurden die bei den Konsilen gestellten Diagnosen und erhobenen Anamnesen von verschiedenen Psychiatern dokumentiert und die Belastung durch COVID-19 ausschließlich entsprechend subjektiver Patientenwahrnehmung erfasst. Dennoch ergeben diese initialen qualitativen Beobachtungen in dem Zeitraum der strengsten Ausgangsbeschränkungen einen weiteren Hinweis auf das Ausmaß psychischer Belastungen infolge von COVID-19. Eine Validierung und Ergänzung der Daten in einem größeren Kollektiv verschiedener Kliniken wäre wünschenswert.

## Fazit für die Praxis

Zusammengefasst hat die COVID-19-Pandemie in den hier untersuchten Stichproben an einer Universitätsklinik nicht zu einer Zunahme der absoluten Zahl von Notaufnahmen wegen psychischer Probleme geführt, aber der Anteil psychiatrischer Diagnosen an Vorstellungen in der Notaufnahme hat sich nahezu verdoppelt. Zudem hat sich das Profil der konsiliarischen Anforderungen in der Krisenversorgung während stationärer Aufenthalte am Beginn der Pandemie mit erhöhter Suizidrate unter COVID-19-belasteten psychiatrischen PatientInnen qualitativ geändert. Höheres Alter und vorbestehende Erkrankungen können bei einer COVID-19-Infektion die Entwicklung von Verwirrtheitszuständen begünstigen. Besuchsverbote im Krankenhaus, so unvermeidbar sie als Bestandteil des Infektionsschutzes auch sein mögen, stellen für PatientInnen mit fortgeschrittenen somatischen Erkrankungen eine erhebliche psychische Belastung dar. Menschen mit Abhängigkeits- und anderen psychischen Vorerkrankungen bereiten die Ausgangsbeschränkungen möglicherweise besondere Schwierigkeiten. Diese Gruppe und jene PatientInnen, welche unter den ersten Auswirkungen der Wirtschaftskrise leiden, weisen in unserer untersuchten Patientengruppe ein hohes Risiko für Suizidversuche auf. Dem muss trotz der Infektionsschutzmaßnahmen mit möglichst niederschwelligen psychiatrischen und ökonomischen Hilfsangeboten Rechnung getragen werden.
